# Benefits and Risks of AI in Health Care: Narrative Review

**DOI:** 10.2196/53616

**Published:** 2024-11-18

**Authors:** Margaret Chustecki

**Affiliations:** 1 Department of Internal Medicine Yale School of Medicine New Haven, CT United States

**Keywords:** artificial intelligence, safety risks, biases, AI, benefit, risk, health care, safety, ethics, transparency, data privacy, accuracy

## Abstract

**Background:**

The integration of artificial intelligence (AI) into health care has the potential to transform the industry, but it also raises ethical, regulatory, and safety concerns. This review paper provides an in-depth examination of the benefits and risks associated with AI in health care, with a focus on issues like biases, transparency, data privacy, and safety.

**Objective:**

This study aims to evaluate the advantages and drawbacks of incorporating AI in health care. This assessment centers on the potential biases in AI algorithms, transparency challenges, data privacy issues, and safety risks in health care settings.

**Methods:**

Studies included in this review were selected based on their relevance to AI applications in health care, focusing on ethical, regulatory, and safety considerations. Inclusion criteria encompassed peer-reviewed articles, reviews, and relevant research papers published in English. Exclusion criteria included non–peer-reviewed articles, editorials, and studies not directly related to AI in health care. A comprehensive literature search was conducted across 8 databases: OVID MEDLINE, OVID Embase, OVID PsycINFO, EBSCO CINAHL Plus with Full Text, ProQuest Sociological Abstracts, ProQuest Philosopher’s Index, ProQuest Advanced Technologies & Aerospace, and Wiley Cochrane Library. The search was last updated on June 23, 2023. Results were synthesized using qualitative methods to identify key themes and findings related to the benefits and risks of AI in health care.

**Results:**

The literature search yielded 8796 articles. After removing duplicates and applying the inclusion and exclusion criteria, 44 studies were included in the qualitative synthesis. This review highlights the significant promise that AI holds in health care, such as enhancing health care delivery by providing more accurate diagnoses, personalized treatment plans, and efficient resource allocation. However, persistent concerns remain, including biases ingrained in AI algorithms, a lack of transparency in decision-making, potential compromises of patient data privacy, and safety risks associated with AI implementation in clinical settings.

**Conclusions:**

In conclusion, while AI presents the opportunity for a health care revolution, it is imperative to address the ethical, regulatory, and safety challenges linked to its integration. Proactive measures are required to ensure that AI technologies are developed and deployed responsibly, striking a balance between innovation and the safeguarding of patient well-being.

## Introduction

Artificial intelligence (AI) has rapidly proliferated across various sectors in recent years, with the health care industry emerging as a primary arena for its transformative potential. This technological advancement holds promise for revolutionizing patient care and administrative operations by leveraging vast longitudinal patient data [1]. AI encompasses a spectrum of technologies, including machine learning (ML), natural language processing (NLP), rule-based expert systems (RBES), physical robots, and robotic process automation, each offering unique capabilities from predictive modeling and disease detection to enhancing surgical precision and automating administrative tasks [2-7]. The integration of AI into health care promises heightened diagnostic accuracy, informed decision-making, and optimized treatment planning, thereby potentially reducing medical errors and improving patient outcomes [1].

However, alongside these promising developments, AI adoption in health care is accompanied by significant ethical and regulatory challenges that require careful consideration [8]. Concerns range from safeguarding patient data privacy to addressing algorithmic biases that may perpetuate disparities in health care outcomes [9,10]. The regulatory landscape is evolving to keep pace with technological advancements, aiming to establish robust governance frameworks that ensure the responsible use of AI in health care settings. Furthermore, the advent of pretrained large language models, exemplified by models like BERT (Bidirectional Encoder Representations from Transformers), GPT (Generative Pre-trained Transformer), and their variants, has further expanded the capabilities of AI in health care [11-14]. These models leverage vast amounts of text data to learn rich representations of language, enabling tasks ranging from clinical documentation improvement to automated summarization of medical literature [15,16].

Against this backdrop, this study presents a narrative review aimed at comprehensively exploring the multifaceted role of AI in health care. By synthesizing existing literature, this research aims to provide insights into the diverse applications of AI, its associated benefits, and the ethical and regulatory considerations that underpin its integration into clinical practice [9,10,17]. This review aims to facilitate informed decision-making among health care professionals, policy makers, and researchers, fostering a balanced approach that maximizes the benefits of AI while mitigating potential risks within the health care landscape.

This review seeks to contribute to ongoing discussions on AI ethics, governance, and effective deployment strategies, thereby guiding the responsible and impactful adoption of AI technologies in health care. By examining current trends, challenges, and future directions, this review aims to lay the groundwork for advancing AI’s role in enhancing health care delivery, improving patient outcomes, and supporting health care systems globally.

## Methods

### Overview

This narrative review aims to assess the benefits and risks associated with the integration of AI into health care, with a primary focus on potential biases, transparency issues, data privacy concerns, and safety risks. A literature review was conducted to explore the current landscape of AI applications in health care and to identify relevant ethical, regulatory, and safety considerations.

### Eligibility Criteria

Specific inclusion and exclusion criteria were established to guide the selection of studies for this narrative review. Studies were included if they were relevant to the 3 core concepts of AI, ethics, and health and were written in the English language. Articles were excluded if they did not explicitly address each of the core concepts of AI, ethics, and health or if they were not written in English. In addition, studies focusing solely on ethics and big data without explicit mention of AI methods or applications were excluded. Non–peer-reviewed academic literature, such as letters and non–peer-reviewed conference proceedings, as well as books and book chapters, were also excluded as they were deemed irrelevant to this review. No restrictions were applied regarding the publication date or study design to ensure a broad overview of the topic.

### Information Sources

The literature search used 8 electronic databases: OVID MEDLINE (1946-present), OVID Embase (1947-present), OVID PsycINFO (1806-present), EBSCO CINAHL Plus with Full Text (1937-present), ProQuest Sociological Abstracts (1952-present), ProQuest Philosopher’s Index (1940-present), ProQuest Advanced Technologies & Aerospace (1962-present), and Wiley Cochrane Library. Search strategies were tailored to each database (Multimedia Appendix 1), using controlled vocabulary, Medical Subject Headings (MeSH) terms, EMTREE terms, American Psychological Association’s Thesaurus of Psychological Index Terms, CINAHL headings, Sociological Thesaurus, Philosopher’s Index subject headings, and Advanced Technologies & Aerospace subject headings. The searches were limited to English language–only articles, and filters excluding animal studies were applied to specific databases. In addition, a filter for health or medicine-related studies was applied to the Advanced Technologies & Aerospace database.

The final searches of the peer-reviewed literature were completed on June 23, 2023. Gray literature was not searched in this narrative review.

### Selection and Sources of Evidence

All identified records from the academic literature searches were imported into the reference management software EndNote (Clarivate). After removing duplicate records, screening was conducted in 2 steps: initial title and abstract screening followed by full-text review. Full-text reviews were conducted to ensure that the selected studies provided substantial insights for the narrative synthesis.

### Data Charting Process

Data charting forms were developed and refined based on the narrative review research question. The forms included fields for recording data such as the objective of each paper, institutional affiliations of authors, publication year, country of the first and corresponding authors, conflict of interest disclosures, health context of interest, AI applications or technologies discussed, ethical concepts, issues or implications raised, reference to global health, and recommendations for future research, policy, or practice. Data were recorded directly into the data charting form with corresponding page numbers to ensure accuracy.

### Synthesis of Results

Data analysis included thematic components. Thematic analysis was conducted inductively, generating open descriptive codes from a sample of records. Codes were applied to relevant data points across all records, with new codes added as needed. These codes were then organized into themes, allowing for the identification of commonalities and gaps in the literature. Results are presented in a narrative format.

## Results

### Overview

Within the realm of integrating AI into health care, this narrative review has revealed a broad range of insights that span a spectrum of possibilities and challenges. This section categorizes the findings into 2 overarching categories: “Benefits” and “Risks.” Each category encapsulates a tapestry of themes that emerged from an exploration of academic literature. As these themes are explored, the multifaceted landscape of AI’s influence on health care is illuminated. The “Benefits” section unveils the potential for AI to revolutionize health care delivery, ushering in more accurate diagnoses, personalized treatment regimens, and streamlined resource allocation. Conversely, the “Risks” section delves into the intricate ethical, privacy, and safety concerns that accompany the integration of AI into clinical settings. Through a comprehensive examination of these themes, this review provides a nuanced perspective on the implications and imperatives in harnessing AI’s potential for the betterment of health care.

**Figure 1:**
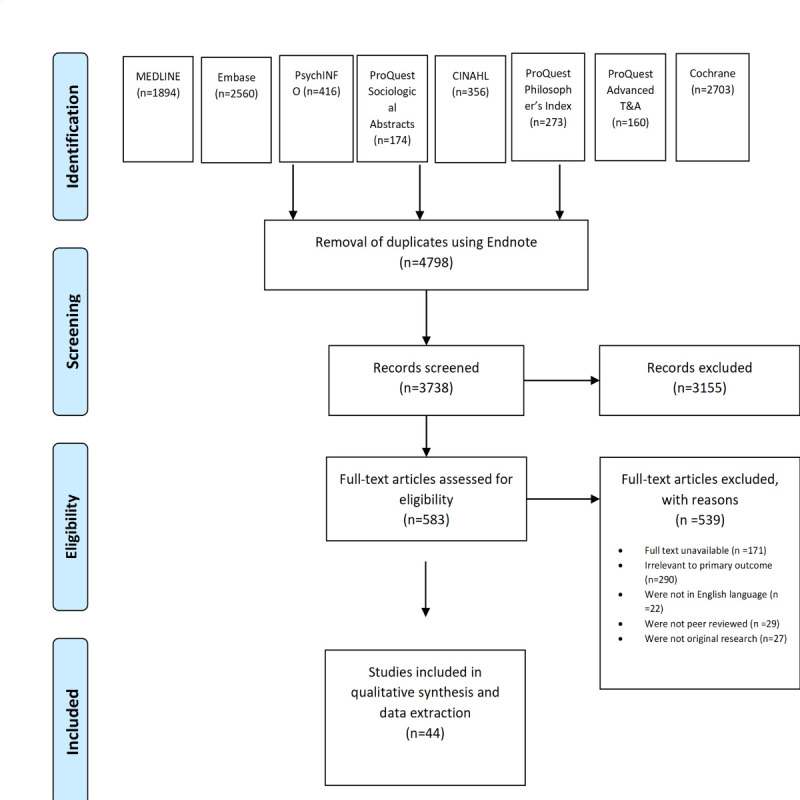
Preferred Reporting Items for Systematic Reviews and Meta-Analyses (PRISMA) study selection flow diagram outlining the literature review process when searching for articles on various databases.

The systematic literature review process, as illustrated in the PRISMA (Preferred Reporting Items for Systematic Reviews and Meta-Analyses) flow diagram (Figure 1), outlines a thorough and rigorous methodology. Initial searches across multiple databases—MEDLINE, Embase, PsycINFO, ProQuest Sociological Abstracts, CINAHL, ProQuest Philosopher’s Index, ProQuest Advanced Technologies & Aerospace, and Cochrane Library—yielded a total of 8796 articles. After removing 4798 duplicates using Endnote, 3738 unique records were screened for relevance. Of these, 3155 articles were excluded based on title and abstract review for not meeting the inclusion criteria. The remaining 583 articles underwent full-text assessment for eligibility. Further evaluation led to the exclusion of 539 articles due to various reasons, such as unavailability of full text (n=171), irrelevance to the primary outcome (n=290), non-English language (n=22), not being peer-reviewed (n=29), and not being original research (n=27). Ultimately, 44 studies were included in the qualitative synthesis and data extraction. This meticulous selection process ensured that the final set of studies provided a robust and representative foundation for examining the integration of AI in health care.

### Benefits of AI in Health Care

Textbox 1 below describes the main benefits of implementing AI in health care. The benefits are explained in detail below.

**Benefits of artificial intelligence (AI) in health care.**

**Medical benefits**

- Helps in prediction of various risks and diseases
- Helps in prevention and control of various diseases
- Leads to better data-driven decisions within the health care system
- Assists in improving surgery
- Supports mental health

**Economic and social benefits**

- Reduction in posttreatment expenditures
- Cost saving through early diagnosis
- Cost saving with enhanced clinical trials
- Patient empowerment
- Relieving medical practitioners’ workload

### Medical Benefits

AI adoption in health care offers various medical, economic, and social benefits. This section discusses some of the key medical benefits of AI.

#### Prediction of Risks and Diseases

AI leverages big data to predict diseases and assess risk exposure among patients. For example, Google collaborates with health delivery networks to develop prediction models that alert clinicians of high-risk illnesses like sepsis and heart failure [18]. ML models can also be used to forecast populations at risk of specific diseases or accidents [19,20]. In addition, AI algorithms, such as deep learning, aid in disease classification and enable more personalized care [21].

#### Prevention and Control of Diseases

AI can play a significant role in the prevention and control of diseases. For instance, AI can enhance sexually transmitted infection (STI) prevention and control by improving surveillance and intervention. By analyzing publicly available social media data, AI can predict county-level syphilis prevalence, enabling faster and more efficient monitoring [22]. AI can also analyze trends in web data to reduce the stigma associated with STI prevention and care and identify and flag STI-related misinformation [22].

#### Data-Driven Decision Making

AI enables better data-driven decisions within the health care system. In a digitalized health care environment, the quality of decision-making relies on the availability and accuracy of underlying data [23]. AI can assist in decision-making by offering real-time recommendations based on clinical guidelines or advancements, reducing the likelihood of medical mistakes [24]. For example, IBM Watson Health uses ML to provide clinical decision support and achieved a high level of agreement with physician recommendations [25].

#### Improvement in Surgery

AI has made significant advancements in surgical procedures. Robotic surgery, such as in gynecologic, prostate, and oral and maxillofacial surgery, enhances surgical precision and predictability [7,26]. Telesurgical techniques driven by AI enable remote surgery and provide better supervision of surgeons [27]. AI-powered surgical mentorship allows skilled surgeons to offer real-time advice and guidance to other surgeons during procedures, improving surgical outcomes [28].

#### Mental Health Support.

AI use in mental health treatments is growing as patients prefer simple and quick feedback [29]. According to Lovejoy et al [30], psychiatric professionals have historically relied on therapeutic discourse and patient narrative to assess mental health since language is the primary means to communicate our emotional and mental well-being. Recent advancements in AI have opened up new perspectives on the subject by enabling technology to infer emotional meaning from more data sources [30]. According to Habermann [31], with a unique combination of NLP and sentiment analysis, data scientists have developed algorithms to comprehend human emotion from the text. Le Glaz et al [32] mentions that in recent years, NLP models have been used to track mental self-disclosure on Twitter, forecast suicide risk online, and identify suicidal thoughts in clinical notes. These models are used in medicine to give complete details about a patient’s emotional and psychological health [31].

### Economic and Social Benefits

In addition to the medical health benefits, using AI in health care has other economic and social advantages, as discussed below.

#### Reduction in Posttreatment Expenditures.

AI-powered systems can analyze posttreatment result patterns and identify the most effective remedies based on patient profiles. This personalized approach to care can significantly reduce the expenses associated with posttreatment complications, which are a major cost driver in health care systems worldwide [33]. By providing immediate diagnosis and appropriate interventions, AI can help minimize the financial burden of posttreatment complications and lead to substantial cost savings.

#### Cost Saving Through Early Diagnosis.

AI has demonstrated superior accuracy and speed in analyzing medical images, such as mammograms, leading to the early detection of diseases like breast cancer. By enabling prompt diagnosis and action before issues escalate, AI can help reduce health care costs associated with late-stage diagnoses [28]. In addition, AI’s ability to process and interpret various medical tests, such as computed tomography scans, with high accuracy reduces the likelihood of physician errors, contributing to cost savings.

#### Cost Saving with Enhanced Clinical Trials.

AI-powered programs can simulate and evaluate numerous potential treatments to predict their effectiveness against various diseases, optimizing the drug development process in clinical trials [34]. By leveraging biomarker monitoring frameworks and analyzing large volumes of patient data, AI accelerates the evaluation of potential treatments, leading to significant cost savings in the development of life-saving medications.

#### Patient Empowerment.

AI has the potential to empower individuals in managing their health. Wearable devices, such as smartwatches, can collect standard health data, which AI algorithms can analyze to provide personalized health recommendations and warnings for potential diseases [35]. Smartphone apps that use ML algorithms can help patients with chronic diseases better manage their conditions, leading to healthier populations and reduced health care expenses [36].

#### Relieving Medical Practitioners’ Workload.

AI technologies can alleviate the burden on health care workers by assisting with administrative tasks, data analysis, and image interpretation. AI can automate clerical responsibilities, analyze patient data more efficiently, and aid in diagnosing various medical conditions [37,38]. By reducing manual labor and prioritizing critical cases, AI helps save time and resources for medical practitioners, ultimately leading to increased productivity and improved patient care.

### Risks of AI in Health Care

The risks of AI in health care are listed in Textbox 2.

**Risks of artificial intelligence (AI) in health care.**

**Risks of AI in health care**

- AI diagnosis is not always superior to human diagnosis
- AI programs may be difficult to understand and overly ambitious
- Implementation issues
- Transparency issues and risks with data sharing
- Biases
- Mistakes in disease diagnosis or AI cannot be held accountable
- Data availability and accessibility
- Regulatory concerns
- Social challenges

#### AI Diagnosis Is Not Always Superior to Human Diagnosis.

While AI has the potential to improve accurate diagnosis, it is not always superior to human diagnosis. Early AI systems, such as the MYCIN program developed in the 1970s, showed promise in diagnosing and treating diseases but did not outperform human diagnosticians [39]. These RBES needed better integration with clinical workflows and medical record systems to be practical and effective. In addition, AI models can suffer from overfitting, generating irrelevant correlations between patient characteristics and outcomes, which can lead to incorrect predictions when applied to new cases [40].

#### Challenges in Understanding and Ambition of AI Programs

Physicians may find it challenging to understand AI programs, particularly in complex domains like cancer diagnosis and treatment. IBM’s Watson program, which combines ML and NLP, garnered attention for its focus on precision medicine. However, integrating Watson into care processes and systems and programming it to handle certain types of cancer has proven difficult [41]. The ambition of AI programs, such as tackling complex cancer therapy, may exceed their current capabilities.

#### Implementation Issues

Implementing AI in health care faces several challenges. RBES embedded in electronic health care systems are commonly used but may lack the accuracy of algorithmic systems based on ML. These RBES struggle to keep up with evolving medical knowledge and handle large amounts of data [42]. The lack of empirical evidence confirming the efficacy of AI-based treatments in prospective clinical trials hinders successful implementation [43]. Much of the AI research in health care is preclinical and lacks real-world validation [44]. Integration into physician workflow is crucial for successful implementation, but there are limited examples of AI integration into clinical treatment, and training physicians to use AI effectively can be a time-consuming process [45].

#### Transparency Issues and Risks With Data Sharing

The use of intelligent machines in health care decision-making raises concerns about accountability, transparency, permission, and privacy [2]. Understanding and interpreting AI systems, such as deep learning algorithms used in image analysis, can be challenging [2]. Physicians who lack comprehension of the inner workings of AI models may struggle to communicate the medical treatment process to patients [46]. Increased reliance on AI may lead to automated decision-making, limiting the contact and communication between health care workers and patients [46].

The rapid emergence of new technologies in health care has sparked skepticism due to the risks associated with data sharing [17]. There is a need for public norms that ensure data governance and openness, as well as improve patient understanding of how and why data are used [17]. Concerns about privacy violations arise from the collection of large data sets and the potential for AI to anticipate personal information [47]. Patients may perceive this as a violation of their privacy, especially if the findings are made public to third parties [48].

Respecting patient confidentiality and acquiring informed consent for data use are ethically required [49]. AI systems should be protected from privacy breaches to prevent psychological and reputational harm to patients [49]. Recent incidents, such as the misuse of Facebook personal data by Cambridge Analytica and the sharing of patient data without explicit consent by the Royal Free London NHS Foundation Trust, have raised concerns about privacy violations [49,50].

#### Biases

ML systems in health care can be prone to algorithmic bias, leading to predictions based on noncausal factors like gender or ethnicity [51]. Prejudice and inequality are among the risks associated with health care AI [28]. Biases present in the data used to train AI systems can result in inaccurate outcomes, especially if certain races or genders are underrepresented [28]. Unrepresentative data can further perpetuate health inequities and lead to risk underestimation or overestimation in specific patient populations [52].

#### Mistakes in Disease Diagnosis and Lack of Accountability

AI systems can make mistakes in patient diagnosis and treatment, creating potential harm [28]. Holding AI systems accountable can be challenging, as liability concerns arise regarding errors and the allocation of responsibility [53]. The lack of explanation from deep learning algorithms can hinder both legal accountability and scientific understanding, potentially eroding patients’ trust in the system [54].

Determining accountability for AI failures is an ongoing challenge, as holding the physician accountable may seem unjust, while holding the developer accountable may be too removed from the clinical setting [24]. The question of who should be held accountable when AI systems fail remains to be answered [24].

#### Data Availability and Accessibility

Large amounts of data from various sources are required to train AI algorithms in health care [55]. However, accessing health data can be challenging due to fragmentation across different platforms and systems [55]. Data availability in health care is limited, and there is often a reluctance to share data between hospitals [56]. The continuous availability of data for ongoing improvement of ML-based systems can be difficult due to organizational resistance [57]. Technological advancements and improved algorithms can help address the problem of limited data sets [57].

#### Regulatory Concerns

The autolearn feature of AI software poses regulatory challenges as algorithms evolve continuously with use [58]. This creates the need for additional policies and procedures to ensure patient safety [58]. Many countries have yet to formalize regulatory guidelines for assessing AI algorithmic safety, which can hinder AI adoption and lead to risky practices [59]. The lack of industry rules on the ethical usage of AI in health care further complicates the accountability issue [60]. Efforts by the Food and Drug Administration and National Health Service to establish guidelines and standards are ongoing but pose barriers to regulatory approval [60,61].

#### Social Challenges

Misconceptions about AI replacing health care jobs lead to skepticism and aversion to AI-based interventions [43]. However, the arrival of AI does not necessarily mean job obsolescence but rather job reengineering [62]. Overcoming skepticism and fostering trust in AI requires a better understanding of its capabilities and meaningful public discourse [62]. Improving public and health care professionals’ understanding of AI is essential to managing expectations and addressing concerns.

## Discussion

### Principal Findings

This narrative review delves into the dynamic landscape of AI integration in health care, aiming to uncover a spectrum of perspectives, concerns, and opportunities. This exploration encompasses a diverse range of health care settings from different countries and regions, unveiling a rich tapestry of AI adoption. Overall, AI offers tremendous potential and will continue to play a crucial role in future health care decisions. If AI is successfully used, it can reduce pressure on health care workers while improving work quality by lowering mistakes and improving precision. It has the potential to give people more control over their health decisions and can lower avoidable hospitalizations. It can broaden the scope of medical knowledge and build on the present clinical guidelines. Given its advantages and capability to drive the development of precision medicine, it is universally acknowledged to be a much-needed enhancement in medicine. AI is anticipated to eventually master most of the essential domains within health care.

However, there are some difficulties associated with incorporating AI in health care. Acquiring enough data to train precise algorithms is a continual effort that necessitates a shift in attitude towards data sharing that promotes technical advancement. Specific guidelines on how to securely adopt and evaluate AI technology and research on AI’s potential and limitations are required. Robust research is also required to empirically demonstrate the benefits of AI use in the actual world. While the perfect conditions for successful AI adoption may not yet exist, there is still room for AI advancement in health care.

Given that AI has tremendous potential and is the future, there are a few crucial points to consider when using AI in health care. First, given the need for more general agreement in AI governance, it may be impossible to develop AI-based systems whose algorithms can be generalized across all health care domains. As a result, it may be wise to concentrate on systems that can be implemented and used effectively in the health care institutions for which they were designed. Fundamentally, patient care must take priority over the excitement of cutting-edge technology. The AI system’s safety and competence must be weighed for use only when appropriate and valuable to patients.

Second, AI in health care must still be complemented with human input. Although AI has advantages in speed and accuracy, physicians are still needed for more cognitively complex or psychological elements and activities. Similarly, although the detection and monitoring of vital disease symptoms are now automated, the objective behind AI is not to eliminate physician input but to focus their expertise on areas where they are more necessary and on what computers cannot and may never imitate. Therefore, focusing on developing complementarity between the use of AI and physicians by training is essential.

In addition, while it is critical to lower expectations, it is also critical not to be excessively gloomy about the role of AI in health care. While physicians may need to comprehend the processes of AI algorithms, most physicians understand magnetic resonance imaging or computed tomography to some level. Despite a lack of individual physician comprehension of their specific process, these studies are extensively used. The lack of transparency in ML algorithms may thus be tolerable if the algorithm’s efficacy can be demonstrated. Again, this can be achieved with training and familiarizing the physician with the AI system.

Rather than putting AI to a standard of either perfect or nil results, one should compare the outcomes of using AI to those of the natural world, where physicians can and will make mistakes. Importantly, AI is dynamic in nature and can improve using more extensive data sets. As a result, it is entirely possible that the combined usage of physician and AI input would be more successful over time. However, it is critical not to overstate the status of AI. Its implementation in health care will be a careful, deliberate, and progressive process, including strict control and monitoring of its use and efficacy. AI can help patients and increase the quality of care when combined with input and oversight from health care professionals. AI systems will not wholly replace human clinicians but will supplement their efforts to care for patients. Human therapists may eventually shift towards jobs and job designs that require distinctly human skills, such as enthusiasm and knowledge to use AI in health care.

As global communities live longer lives and the prevalence of chronic disease rises, the rising cost of health care will remain a hot topic among health care stakeholders. It is time to seek the assistance of machines as they can potentially reduce economic costs. Furthermore, coordination between government and private sector industry partners is vital to realize this potential and take advantage of AI’s full potential in health care delivery.

With all this, the key challenge for AI in many sectors of health care is ensuring its adoption in daily clinical practice rather than whether the technologies will be equipped to be effective. AI systems must be approved by regulators, linked with electronic health record systems, standardized to the point that similar products perform similarly, taught to medical practitioners, paid for by public or private payer organizations, and modified in the field over time for widespread adoption to occur shortly. Since AI has a significant and lasting impact on lives and is the future of health care, it is essential to address the associated concerns. Given its importance, AI needs proper policy guidelines and regulations regarding its usage in health care to reap its maximum benefits.

### Comparison With Previous Literature

In comparing the findings of this review with existing literature, several key similarities and differences emerge. This review aligns with Gazquez-Garcia et al [63] and Mooghali et al [64] in highlighting the crucial role of health care professional training for effective AI integration. Both emphasize the need for proficiency in AI fundamentals, data analytics, and ethical considerations, reinforcing the notion that successful AI adoption requires a well-prepared workforce. The review also echoes Sapci and Sapci’s [65] advocacy for incorporating AI education into medical curricula to address future challenges.

However, this review diverges in its emphasis on practical AI implementation challenges. While Moghadasi et al [66] and Muley et al [67] discuss the risks associated with AI, including the need for enhanced transparency and stakeholder collaboration, this review adds a nuanced perspective on balancing AI’s potential benefits with its ethical risks. For instance, this review highlights the importance of human oversight and the complementarity of AI with clinician expertise, which aligns with Morley et al [68] and Zhang and Zhang [69] but also offers additional insights into practical implementation issues not fully covered in the previous reviews.

In terms of public perception, this review supports Kerstan [70] and Castagno and Khalifa [71] by acknowledging that trust in AI is influenced by knowledge and transparency. However, it further explores the dynamic interaction between AI’s promise and the necessity for rigorous validation and ethical governance, as discussed by Macrae [72] and Tulk Jesso et al [73]. This review underscores that while AI has the potential to revolutionize health care, its integration must be handled with careful consideration of both practical and ethical dimensions to achieve meaningful improvements in patient care and outcomes.

### Strengths and Limitations

First, the generalizability of the findings may be affected by the inherent variations in study methodologies, AI implementations, and health care settings across different regions. This heterogeneity introduces variability that could influence the applicability of the conclusions. To mitigate this limitation, rigorous search strategies were used across multiple databases to include a diverse range of studies. Future reviews could benefit from incorporating more standardized studies to enhance generalizability. Second, the reliance on published literature from electronic databases introduces potential publication bias. Studies with positive outcomes related to AI integration in health care may be more likely to be published, which could skew perceptions of AI effectiveness and adoption rates. Efforts were made to address this bias by including a broad range of databases and emphasizing recent literature. Future research should aim to include unpublished studies or grey literature to provide a more balanced view. Third, the rapid evolution of AI technologies means that newer developments and implementations may not have been fully captured in this review. The review focused on the most current literature available at the time of the search to address this issue. Regular updates will be necessary to incorporate the latest advancements and ensure the review remains relevant. In addition, the absence of details around stakeholder engagement could have enriched the study by providing additional depth and perspective. Engaging stakeholders in such a dynamic field would offer diverse viewpoints and further validate the conclusions. Future research should consider incorporating stakeholder engagement to enhance the robustness and applicability of the findings.

Despite these limitations, this review offers several notable strengths. It provides a comprehensive overview of AI integration in health care, leveraging rigorous search strategies across multiple databases to ensure a diverse and current collection of literature. This approach contributes to a nuanced understanding of AI’s potential and limitations. Furthermore, the emphasis on recent developments helps ensure that the review reflects the most current trends and advancements in AI technologies.

### Future Directions

Moving forward, further research in the field of AI integration in health care should address several key areas to advance understanding and application. First, studies should prioritize incorporating stakeholder engagement, including health care providers, patients, policymakers, and technology developers, to provide diverse perspectives on AI adoption and implementation strategies, enhancing relevance and acceptance in clinical practice. Second, longitudinal studies are crucial to assess the long-term impacts of AI technologies in health care settings, providing insights into sustainability, scalability, and real-world effectiveness over time. Third, comprehensive research focusing on the ethical implications of AI, including data privacy, algorithm bias, patient consent, and regulatory frameworks, is needed to build trust and ensure responsible deployment. In addition, comparative effectiveness research comparing AI-assisted interventions with standard care protocols can provide evidence of AI’s impact on clinical outcomes, patient safety, and health care efficiency. Interdisciplinary collaboration between computer scientists, health care professionals, social scientists, and ethicists is essential to foster innovative approaches aligned with health care needs. Education and training programs for health care professionals on AI technologies will ensure proficiency in interpreting AI-generated insights and integrating them into patient care effectively. Finally, research should explore how AI can reduce health care disparities and improve access to quality care, particularly in underserved communities and low-resource settings. Addressing these priorities will realize AI’s potential in transforming health care delivery and improving patient outcomes globally.

### Conclusions

In summary, AI presents a transformative force in health care, with the potential to enhance patient care, reduce errors, and broaden medical knowledge. However, its successful integration requires adaptability; complementarity with human expertise; transparency; and a deliberate, incremental approach. AI’s impact on health care is evolutionary, not revolutionary, and collaboration between stakeholders, standardization, education, and robust policies are essential to harness its full potential while upholding patient-centric care and innovation.

## Appendix

Multimedia Appendix 1 Detailed search strategy across databases for artificial intelligence (AI) integration in health care.

## Abbreviations

AI: artificial intelligence
BERT: Bidirectional Encoder Representations from Transformers
GPT: Generative Pre-trained Transformer
MeSH: Medical Subject Headings
ML: machine learning
NLP: natural language processing
PRISMA: Preferred Reporting Items for Systematic Reviews and Meta-Analyses
RBES: rule-based expert systems
STI: sexually transmitted infection

## Data Availability

All data generated or analyzed during this study are included in this published paper and its supplementary information files.
