# Supplementary material for: Benefits and Risks of AI in Health Care: Narrative Review
Source: Interact J Med Res. 2024 Nov 18;13:e53616. doi: 10.2196/53616 (PMC11612599; doi:10.2196/53616)
Supplement: Multimedia Appendix 1 [file ijmr_v13i1e53616_app1.docx]

**Multimedia Appendix 1.** Detailed search strategy across databases for AI integration in health care.

| **Database** | **Search Terms** |
| --- | --- |
| OVID Medline | ("artificial intelligence" OR AI) AND (healthcare OR "medical care" OR medicine) |
| OVID Embase | ("machine learning" OR "deep learning") AND (healthcare OR "medical care" OR medicine) |
| OVID PsychINFO | (AI OR "deep learning" OR "natural language processing") AND (medicine OR health) |
| EBSCO CINAHL Plus | ("natural language processing" OR "machine learning" OR AI) AND (healthcare OR nursing) |
| ProQuest Sociological Abstracts | AI AND ("patient care" OR "clinical decision") |
| ProQuest Philosopher’s Index | ("machine learning" OR "deep learning") AND healthcare |
| ProQuest Advanced Technologies & Aerospace | ("machine learning" OR "deep learning") AND healthcare |
| Wiley Cochrane Library | (("artificial intelligence" OR AI) AND healthcare) |
